# Visualizing the Recruitment Funnel: Results from A National Survey of U.S. Older Adults on Intention to Join an Alzheimer’s Secondary Prevention Trial and Intention to Ask A Study Partner

**DOI:** 10.1002/alz70859_097459

**Published:** 2025-12-25

**Authors:** Emily A. Largent, Josh D Grill, Jason Karlawish, Amy Bleakley

**Affiliations:** ^1^ University of Pennsylvania, Philadelphia, PA USA; ^2^ University of California, Irvine, Irvine, CA USA; ^3^ University of Pennsylvania Perelman School of Medicine, Philadelphia, PA USA; ^4^ University of Delaware, Newark, DE USA

## Abstract

**Background:**

It is important to recruit representative samples into secondary prevention trials to better understand and more effectively treat preclinical Alzheimer’s disease. Past studies have examined barriers to and facilitators of participation in such trials and suggest that the requirement that participants enroll with a knowledgeable informant or “study partner” may disproportionately limit enrollment of individuals from underrepresented racial and ethnic groups.

**Method:**

A national survey of U.S. adults aged 65+ and oversampling Black individuals assessed intention to enroll in a hypothetical secondary prevention trial, the HEALTHY MIND Study. Among those intending to enroll, intention to ask someone to serve as a study partner was also measured. Intention was assessed on a 5‐point scale (very unlikely to very likely); scores above the midpoint were considered representative of intention to act. Descriptive statistics were calculated on all measures for the full sample as well as for those who intended to enroll and those who intended to ask a study partner. Differences were calculated between persons who (1) intended to enroll and those who did not and (2) intended to ask a study partner and those who did not.

**Result:**

In the full sample (n=603), intention to enroll in the HEALTHY MIND Study was low (M: 2.37, SD: 1.31). Among those intending to enroll (24%, n=168), the mean intention to ask someone to be a study partner was 3.45 (SD 1.44). Sixty‐five respondents who expressed an intention to enroll subsequently indicated that they did not intend to ask a study partner. On average, participants who intended to ask a study partner (61%, n=103) identified 2.14 (SD 1.12) individuals they would be willing to ask. There were no significant differences between Black and White respondents in intention to enroll or to ask a study partner.

**Conclusion:**

Our study suggests the study partner requirement is a barrier to recruitment in secondary prevention trials; however, underrepresentation of Black older adults is not attributable to lower intention to participate or to ask a study partner. Instead, lack of representation is likely attributable to other causes such as restrictive inclusion criteria or structural barriers to participation.

